# Nuclear imaging for localization and surgical outcome prediction in epilepsy: A review of latest discoveries and future perspectives

**DOI:** 10.3389/fneur.2022.1083775

**Published:** 2022-12-16

**Authors:** Chanan Sukprakun, Supatporn Tepmongkol

**Affiliations:** ^1^Division of Nuclear Medicine, Department of Radiology, Faculty of Medicine, Chulalongkorn University, Bangkok, Thailand; ^2^Chulalongkorn University Biomedical Imaging Group (CUBIG), Faculty of Medicine, Chulalongkorn University, Bangkok, Thailand; ^3^Chula Neuroscience Center, King Chulalongkorn Memorial Hospital, Bangkok, Thailand; ^4^Cognitive Impairment and Dementia Research Unit, Faculty of Medicine, Chulalongkorn University, Bangkok, Thailand

**Keywords:** epilepsy, nuclear medicine, SPECT, PET, radiopharmaceuticals, techniques, presurgical localization

## Abstract

**Background:**

Epilepsy is one of the most common neurological disorders. Approximately, one-third of patients with epilepsy have seizures refractory to antiepileptic drugs and further require surgical removal of the epileptogenic region. In the last decade, there have been many recent developments in radiopharmaceuticals, novel image analysis techniques, and new software for an epileptogenic zone (EZ) localization.

**Objectives:**

Recently, we provided the latest discoveries, current challenges, and future perspectives in the field of positron emission tomography (PET) and single-photon emission computed tomography (SPECT) in epilepsy.

**Methods:**

We searched for relevant articles published in MEDLINE and CENTRAL from July 2012 to July 2022. A systematic literature review based on the Preferred Reporting Items for Systematic Reviews and Meta-Analysis was conducted using the keywords “Epilepsy” and “PET or SPECT.” We included both prospective and retrospective studies. Studies with preclinical subjects or not focusing on EZ localization or surgical outcome prediction using recently developed PET radiopharmaceuticals, novel image analysis techniques, and new software were excluded from the review. The remaining 162 articles were reviewed.

**Results:**

We first present recent findings and developments in PET radiopharmaceuticals. Second, we present novel image analysis techniques and new software in the last decade for EZ localization. Finally, we summarize the overall findings and discuss future perspectives in the field of PET and SPECT in epilepsy.

**Conclusion:**

Combining new radiopharmaceutical development, new indications, new techniques, and software improves EZ localization and provides a better understanding of epilepsy. These have proven not to only predict prognosis but also to improve the outcome of epilepsy surgery.

## Introduction

One of the most common neurological disorders is epilepsy. Approximately, one-third of patients with epilepsy have seizures refractory to antiepileptic drugs, so-called drug-resistant epilepsy, and further require surgical removal of the epileptogenic region (1). The epileptogenic zone (EZ) is defined by Lüders et al. as “the minimum amount of cortex that must be resected (inactivated or completely disconnected) to produce seizure freedom” (2). Thus, by this definition, EZ can be defined after surgery. To make a decision for epilepsy surgery, patients must undergo a series of tests as part of the routine standard preoperative evaluation to identify the “presumed” epileptogenic zone and determine surgical candidates. There are several non-invasive methods for this purpose, such as history taking, neurological examination, neuropsychological evaluation, electroencephalogram (EEG), magnetic resonance imaging (MRI), single-photon emission computed tomography (SPECT) using either ^99m^Tc-ethyl cysteinate dimer (ECD) or ^99m^Tc-hexamethyl propylene amine oxime (HMPAO), positron emission tomography (PET) using [^18^F]-fluorodeoxyglucose (FDG), and an invasive method, that is, intracranial EEG (iEEG) as electrocorticography (ECoG) using subdural grid electrodes or stereotactic EEG (SEEG) using depth electrodes (3). For EZ localization, no single test is ideal for identification, thereby it needs the consensus of multiple investigations.

Several cases of unfavorable surgical outcomes are still observed, although we have several sophisticated imaging modalities and techniques for this evaluation. This suggests the need for additional radiotracer and more advanced image analysis techniques or software for better EZ localization that provides a post-surgical seizure-free outcome. As a result, there have been increasing research studies in the field of PET and SPECT in epilepsy recently.

In this review, we provide the latest discoveries, current challenges, and future perspectives in the field of PET and SPECT in epilepsy in the last decade.

## Methods

### Search methods and selection criteria

**Figure 1:**
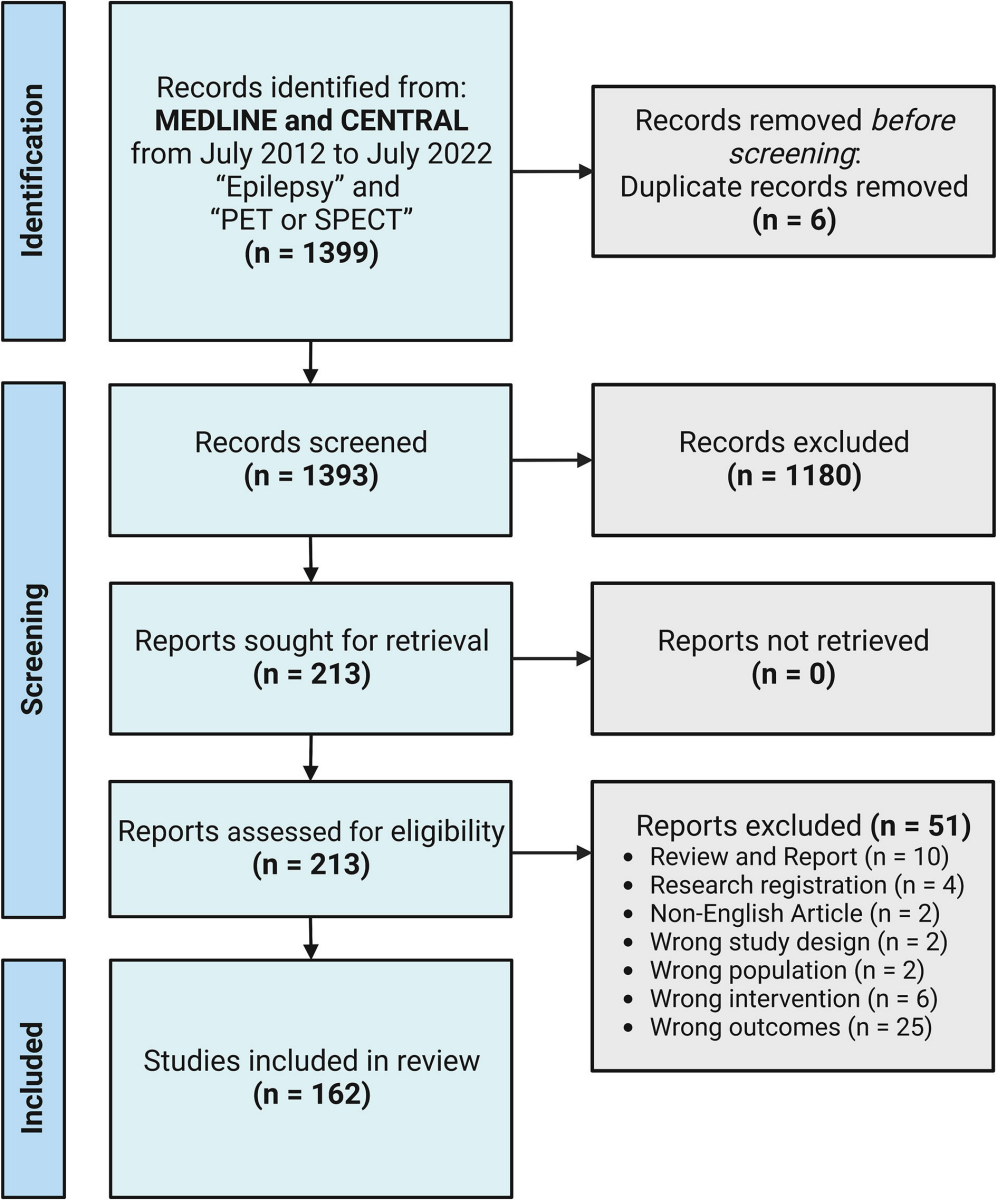
PRISMA flow diagram for the study recruitment. A final 162 studies were included.

In July 2022, we searched the relevant articles published in MEDLINE and CENTRAL from July 2012 to July 2022. A systematic literature review based on the Preferred Reporting Items for Systematic Reviews and Meta-Analysis (PRISMA) was conducted. First, we used the keywords “Epilepsy” and “PET or SPECT,” which yielded 1,399 studies imported for the title and abstract screening with six duplicates removed using Covidence (systematic review software, Veritas Health Innovation, Melbourne, Australia; available at www.covidence.org). We included both prospective and retrospective studies using PET or SPECT scans in patients with epilepsy. Studies with preclinical subjects or not focusing on EZ localization or surgical outcome prediction using recently developed PET radiopharmaceuticals, novel image analysis techniques, or new software were excluded from the review. Reviews and case reports were also excluded. After this step, there were 1,180 irrelevant studies. Of the 213 full-text studies assessed, 51 studies were further excluded. The final analysis of 162 studies was reviewed and categorized as the following: recent findings and development of non-FDG PET radiotracers (*n* = 42) and image analysis techniques or software for the detection of EZ and prediction of surgical outcomes (*n* = 120). PRISMA flow diagram is shown in Figure 1.

## Results

### I. Radiopharmaceuticals

**Figure 2:**
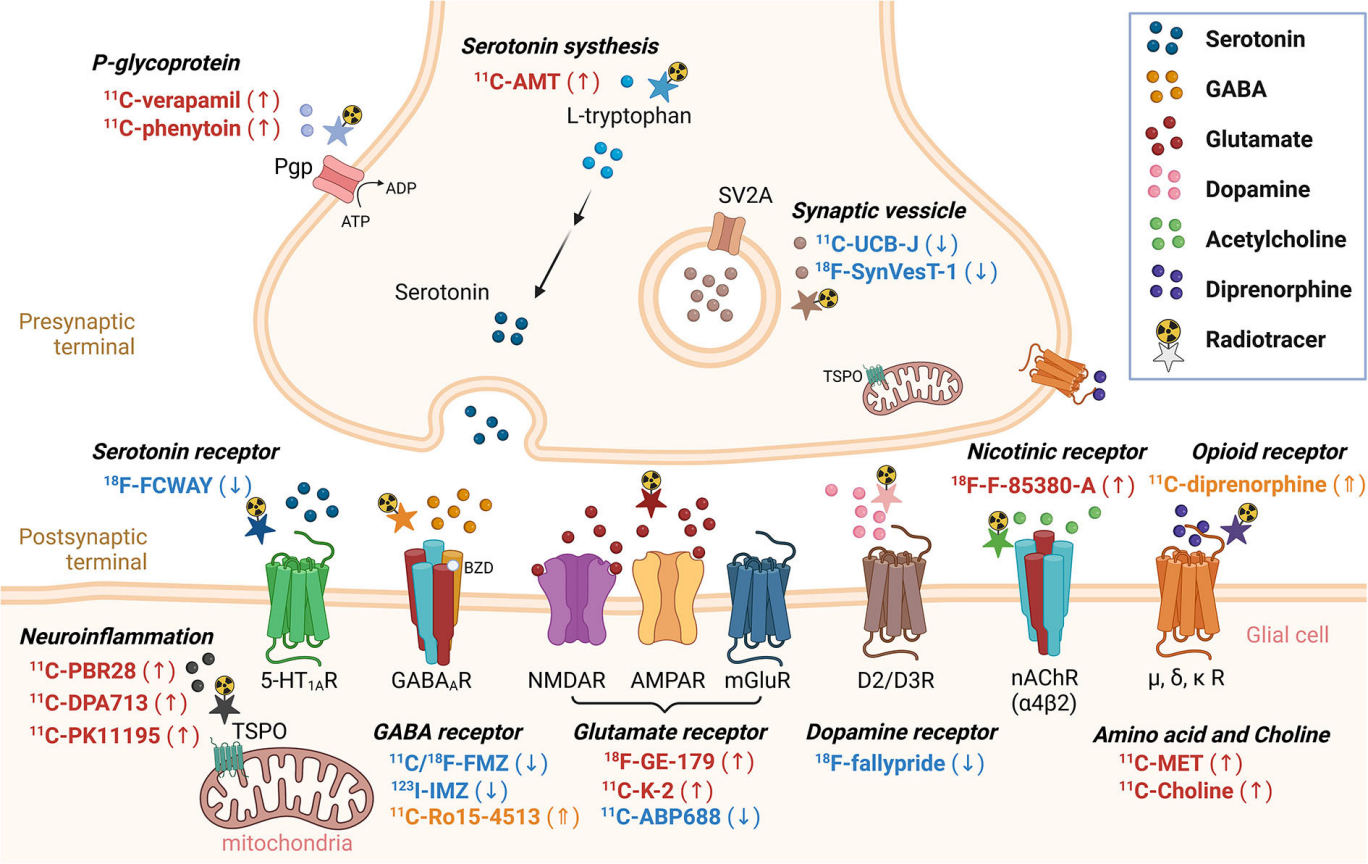
Non-FDG radiotracers used in epilepsy in the last decade. Radiotracers with a downward arrow, upward arrow, or upward double arrow showed decreased uptake, increased uptake, or increased volume of distribution, respectively.

This section summarizes recent findings or developments of non-FDG radiotracers that bind to various receptors or proteins, which were published in the last decade. [^18^F]-FDG, a positron emission radiotracer, is one of the most commonly used radiopharmaceuticals for the detection of the functional deficit zone in patients with drug-resistant epilepsy. Although brain glucose hypometabolism is often observed in the interictal state, it represents both EZ and other functional deficit zones. Thus, to depend on FDG alone is not enough for EZ localization. Several non-FDG radiotracers targeting various specific receptors in an interictal phase were recently proposed for better EZ localization (Figure 2, Table 1).

**Table 1:** Overview of non-FDG radiotracers recently used in epilepsy.

**Group**	**Target**	**Radiotracer**	**Uptake**	**Subjects**	**Main objectives**
Serotonin	Serotonin synthesis	[^11^C]- AMT	Increase	TSC	Localization, lateralization, microRNA expression
	5-HT_1A_ receptor	[^18^F]-FCWAY	Decrease	TLE	Localization, prediction
GABA	GABA_A_ receptor	[^11^C]/[^18^F]-FMZ	Decrease	TLE, ETE	Localization, prediction
		[^123^I]-IMZ	Decrease	TLE, ETE	Localization, prediction
		[^11^C]-Ro15-4513	Increase^*^	TLE	Differentiation from HC
Glutamate	NMDA receptor	[^18^F]GE-179	Increase	TLE, ETE	Differentiation from HC
	AMPA receptors	[^11^C]-K-2	Increase	TLE	Localization
	mGluR5	[^11^C]-ABP688	Decrease	FCD, TLE	Differentiation from HC, lateralization, localization
Dopamine	D2/D3 receptor	[^18^F]-fallypride	Decrease	TLE with HS	Differentiation from HC, localization
Nicotinic cholinergic	Nicotinic acetylcholine receptor	[^18^F]-F-85380-A	Increase	ADNFLE, IGE	Differentiation from HC and focal epilepsy
Opioid	μ, δ, κ receptor	[^11^C]-diprenorphine	Increase^*^	TLE	Differentiation from HC (after PVE correction)
Synaptic vesicle	SV2A	[^11^C]UCB-J	Decrease	TLE	Localization
		[^18^F]-SynVesT-1	Decrease	FCD	Localization
Drug transport	P-glycoprotein	[^11^C]-verapamil	Increase	TLE, ETE	Localization, prediction
		[^11^C]-phenytoin	Increase	HC	
Inflammation	TSPO	[^11^C]PK11195	Increase	FLE	Differentiation from HC
		[^11^C]PBR28	Increase	TLE, ETE	Differentiation from HC, localization
		[^11^C]DPA713	Increase	TLE, ETE	Differentiation from HC, localization
Amino acid	Protein synthesis	[^11^C]-methionine	Increase	DNT, TLE	Differentiation of DNT and other epileptic brain neoplasm, AE with/without neoplasm
Choline	Phospholipid synthesis	[^11^C]-choline	Increase	Insular epilepsy	Localization

ADNFLE, autosomal dominant nocturnal frontal lobe epilepsy; AE, amygdala enlargement; DNT, dysembryoplastic neuroepithelial tumors; ETE, extra-temporal lobe epilepsy; FCD, focal cortical dysplasia; HC, healthy control; HS, hippocampal sclerosis; IGE, idiopathic generalized epilepsy; PVE, partial volume effect; TLE, temporal lobe epilepsy; TSC, tuberous sclerosis complex;

* , increased volume of distribution.

#### 1. Serotonin system

##### 1.1. [^11^C]-AMT and 1.2 [^18^F]-FCWAY

L-tryptophan is an amino acid precursor of the neurotransmitter serotonin. Alpha-[^11^C]-methyl-L-tryptophan ([^11^C]-AMT), a tryptophan analog, reflects the serotonin synthesis rate. [^11^C]-AMT PET shows increased uptake in the EZ and is mainly used for the localization or lateralization of the epileptogenic tubers in patients with tuberous sclerosis complex. Studies have revealed a correlation between AMT uptake and seizure duration or microRNA expression (4–6).

[^18^F]-trans-4-fluoro-N-2-[4-(2-methoxyphenyl) piperazin-1-yl]ethyl]-N-(2-pyridyl) cyclohexanecarboxamide ([^18^F]-FCWAY) is a selective antagonist of 5-hydroxytryptamine receptor 1A (5-HT_1A_). [^18^F]-FCWAY PET shows reduced binding ipsilateral to the epileptic focus in patients with temporal lobe epilepsy (TLE). The asymmetry index (AI) of [^18^F]-FCWAY binding may be used to predict temporal lobectomy outcome (7).

#### 2. GABA receptors

##### 2.1. [^11^C]-FMZ, 2.2 [^18^F]-FMZ, 2.3 [^123^I]-IMZ, and 2.4 [^11^C]-Ro15-4513

For PET and SPECT in epilepsy, various labeled radiotracers for selective antagonists of GABA_A_/benzodiazepine receptor have been used, which showed decreased binding reflecting the neuronal loss in the EZ. C-11 or F-18 labeled flumazenil (FMZ) as PET tracers for either PET or binding potential (BP) images can be used for EZ localization (8, 9). In patients with TLE, the volume of decreased FMZ binding showed no correlation with post-surgical outcomes. However, periventricular white matter [^11^C]-FMZ binding might be used to predict poor surgical outcomes (10, 11). A meta-analysis of [^11^C]-FMZ PET showed an overall sensitivity of 0.62 (95% CI: 0.49–0.73) and a specificity of 0.73 (95% CI: 0.59–0.84) for the localization of the EZ (12). [^18^F]-FMZ PET and [^18^F]-FMZ BP images could be used for EZ localization in TLE complementary to [^18^F]-FDG (13, 14). [^123^I]-iomazenil (IMZ), a SPECT tracer, other than showing reduced binding in the EZ (15–17), the reduction was also observed to extend from the mesial temporal lobe to the ipsilateral extrafocal region in TLE (16). IMZ SPECT correctly localized the seizure onset zone more often than the other two conventional tracers (ECD and IMP), but the lateralization performance was not significantly different from IMP SPECT (18).

[^11^C]-Ro15-4513 is an α5 subunit-selective inverse agonist of GABA_A_ receptor. α5 subunit is a tonic inhibitor, unlike FMZ and IMZ, which are mainly indicative of the distribution of α1 subunit and have shown decreased binding in EZ. [^11^C]-Ro15-4513 parametric images of the volume of distribution showed increased binding in the anteromedial and lateral temporal lobe ipsilateral to the EZ, reflecting shifts of α1 to α5 in TLE with normal MRI and comorbid memory impairment (19).

#### 3. Glutamate receptors

##### 3.1. Ionotropic glutamate receptor

NMDA receptors.

##### 3.1.1. [^18^F]GE-179

[^18^F]GE-179 is a ligand, which binds the phencyclidine recognition site within N-methyl-D-aspartate (NMDA) ion channel. [^18^F]GE-179 PET showed increased NMDA receptor ion channel activation in patients with focal epilepsy compared with healthy controls (20, 21).

AMPA receptors.

##### 3.1.2. [^11^C]-K-2

4-[2-(phenylsulfonylamino)ethylthio]-2,6-difluoro-phenoxyacetamide radiolabeled with ^11^C ([^11^C]-K-2) is a ligand that binds to α-amino-3-hydroxy-5-methyl-4-isoxazole propionic acid (AMPA) receptor. [^11^C]-K-2 PET shows the density of cell surface AMPA receptors. There was increased [^11^C]-K uptake in the EZ of patients with mesial TLE (mTLE) (22, 23).

##### 3.2. Metabotropic glutamate receptor

###### 3.2.1. [^11^C]-ABP688

3-(6-methyl-pyridin-2-ylethynyl)-cyclohex-2-enone-O-^11^C-methyloxime ([^11^C]-ABP688) is a ligand that selectively binds the allosteric site of metabotropic glutamate receptor type 5 (mGluR5) and was used to study network integration. [^11^C]-ABP688 BP images showed decreased mGluR5 availability in the EZ of patients with focal cortical dysplasia and mTLE, reflecting less network integration in both diseases (24–27).

#### 4. Dopamine receptor

##### 4.1. [^18^F]-fallypride

Fallypride is an antagonist of the dopamine D2/D3 receptor. [^18^F]-fallypride PET reflects striatal and extrastriatal D2/D3-receptor binding. In patients with TLE and hippocampal sclerosis, [^18^F]-fallypride BP images showed decreased D2/D3 receptor available region that corresponds to “the irritative zone” surrounding the EZ (28).

#### 5. Nicotinic receptor

##### 5.1. [^18^F]-F-85380-A

[^18^F]-F-85380-A is a ligand that binds to the α4β2 subunit of neuronal nicotinic acetylcholine receptors (nAChRs). Using [^18^F]-F-85380-A PET, the study showed that there was a neurochemical correlation between the peripheral autonomic nervous system and central autonomic nervous system at the dorsal anterior cingulate cortex (dACC) and the anterior insula that represents the salience network (29). Another study by the same group also demonstrated that there was an increased availability of nAChRs in the bilateral dACC of idiopathic generalized epilepsy compared with patients with focal epilepsy or controls (30).

#### 6. Opioid receptor

##### 6.1. [^11^C]-diprenorphine

[^11^C]-diprenorphine is a non-selective antagonist of opioid receptors. Postictal [^11^C]-diprenorphine PET with partial volume effect correction showed increased [^11^C]-diprenorphine volume of distribution relative to the interictal state in the ipsilateral parahippocampal gyrus of patients with TLE (31).

#### 7. Other

##### 7.1. Synaptic vesicle

###### 7.1.1. [^11^C]UCB-J AND 7.1.2 [^18^F]-SYNVEST-1

[(R)-1-[(3-(11C-methyl-11C)pyridin-4-yl)methyl]-4-(3,4,5-trifluorophenyl)pyrrolidin-2-one] or [^11^C]UCB-J is a ligand for synaptic vesicle glycoprotein 2A (SV2A), an integral transmembrane glycoprotein localized to secretory vesicles and is the binding site for some antiepileptic drugs (AEDs). [^11^C]UCB-J PET and [^11^C]UCB-J BP images reflect synaptic density and showed a reduction in the EZ of patients with TLE (32, 33). [^18^F]-SynVesT-1, previously called [^18^F]-SDM-8, is another ligand for SV2A. The loss of SV2A is also related to epileptogenesis in focal cortical dysplasia type II. The EZ of focal cortical dysplasia type II patients had significantly reduced [^18^F]-SynVesT-1 uptake compared with contralateral regions and showed a smaller region of abnormality than [^18^F]-FDG-PET (34).

##### 7.2. P-glycoprotein

###### 7.2.1. [^11^C]-verapamil and 7.2.2 [^11^C]-phenytoin

Verapamil, a calcium channel blocker, is usually used as AED adjunctive therapy. [^11^C]-verapamil is a radiolabeled P-glycoprotein (Pgp) substrate. Overexpression of Pgp is believed to be a possible cause of drug-resistant epilepsy. [^11^C]-verapamil PET showed changes in Pgp function before and after temporal lobectomy, which might be associated with good seizure control (35). Quantitative analysis based on [^11^C]-verapamil PET with Pgp inhibitor, for example, cyclosporin A and tariquidar, could be used for localizing or lateralizing the EZ (36, 37). Phenytoin is another widely used AED. [^11^C]-phenytoin PET is also used for mapping regional Pgp overexpression. A quantification method for kinetic evaluation in healthy controls was developed (38).

##### 7.3. Neuroinflammation

###### 7.3.1. [^11^C]PK11195, 7.3.2 [^11^C]PBR28, and 7.3.3 [^11^C]DPA713

Upregulated translocator protein 18 kDa (TSPO) in response to glial cell activation is a marker of neuroinflammation. TSPO PET with [^11^C]PK11195, the first-generation ligand, showed a region of increased TSPO expression corresponding to clinically defined frontal lobe epilepsy and a postictal scan showing significantly greater inflammation intensity and spatial extent (39). [^11^C]PBR28 and [^11^C]DPA713 are the second-generation ligands of TSPO. [^11^C]DPA713 PET showed increased uptake in the EZ of child-onset epilepsy (40). However, both [^11^C]PBR28 and [^11^C]DPA713 may show increased uptake both on the ipsilateral and contralateral sides of seizure foci in patients with neocortical epilepsy and TLE (41, 42). This may limit the use of these tracers for EZ localization.

##### 7.4. Amino acid and choline

###### 7.4.1. [^11^C]-methionine and 7.4.2 [^11^C]-choline

[^11^C]-methionine is an amino acid tracer, which is widely useful for detecting brain tumors. [^11^C]-methionine PET could differentiate between dysembryoplastic neuroepithelial tumors (DNTs), a much more benign tumor, and other epileptogenic brain neoplasms (43). [^11^C]-methionine PET could detect increased uptake in the enlarged amygdala associated with neoplasms but showed no uptake in non-neoplastic lesions in TLE patients with amygdala enlargement (44).

[^11^C]-choline is an endogenous substrate tracer, which is widely used in primary prostate cancer and low-grade astroglioma. [^11^C]-choline uptake is also increased in epileptogenic low-grade glioma and malformation of cortical development (45).

### II. Image analysis techniques and software

#### Brain perfusion SPECT

The two most commonly used cerebral blood flow agents in epilepsy, ^99m^Tc-ECD and ^99m^Tc-HMPAO, are usually used for brain perfusion SPECT in both the ictal and interictal phases. Regional cerebral blood flow (rCBF) to the seizure onset zone is increased by up to 300% during the ictal phase (46). Therefore, the region of hyperperfusion observed on the ictal SPECT is suspected to be the seizure onset zone. However, ictal SPECT requires rapid injection of the radiotracer during a seizure to capture the seizure onset zone, not the seizure spreading zone. In cases with delayed injection, false localization of the seizure onset zone could be observed. Injection time is defined as the time from the seizure onset to the end time of injection. The longer the injection time, the more false detection of the seizure onset zone in TLE (47). Latency time is defined as the time from the seizure onset to the initiation time of injection. A remote-controlled automated injector was introduced to enhance rapid injection with the additional benefit of less radiation to the staff. Remote-controlled automatic injectors for ictal SPECT showed shorter latency time, a higher detection rate of EZ, and a lower number of patients with repeated studies than the traditional manual injection in pediatric patients (48, 49). Remote-controlled automated injectors for adult patients also showed equivalent injection time (50) or shorter latency time (51) than the traditional manual injection. An EEG-driven autonomous injection system prototype without manual intervention was proposed in the seizure prediction model and showed promising results (52). During the interictal phase, rCBF may show decreased or normal perfusion in the seizure onset zone. Visual comparison of ictal and interictal SPECT to detect hyperperfusion changes and computer-assisted subtraction techniques are routinely performed. Visual analysis of specific findings or parameters is helpful in epilepsy evaluation. Combined ^99m^Tc-ECD SPECT parameters (maximum perfusion on ictal phase combined with maximum perfusion change between ictal and interictal phases) provided more specificity, positive predictive value (PPV), and accuracy than a single SPECT parameter for assessing seizure onset zone in patients with MRI-negative extratemporal lobe epilepsy (53). Relatively increased ictal perfusion in the contralateral central structures, that is, the corpus callosum, basal ganglia, and thalamus, were significantly associated with good surgical outcomes in patients with drug-resistant epilepsy (54).

Computer-assisted subtraction technique, that is, subtraction ictal SPECT coregistered to MRI (SISCOM), is one of the most common techniques performed nowadays. The ictal SPECT images are subtracted by the interictal images to generate the different images. The different or subtraction images were converted into Z-score images using the mean and standard deviation of the differences in all brain voxels. Proper selection of a Z-score (or threshold) is crucial for optimal interpretation. SISCOM with a Z-score of 1.5 or 2.0 is often used. In one study with a 6-month follow-up, SISCOM with a Z-score of 1.5 showed higher sensitivity and specificity for EZ localization than the Z-score of 2.0 (55). Another larger study stated that SISCOM with a Z-score of 1.5 and 2.0 had no significant differences in EZ localization, and inter-observer agreement was higher in the 2.0 threshold (56). When MRI is not available for coregistration, subtraction ictal SPECT coregistered to interictal SPECT (SISCOS) may be used as a makeshift. SISCOM had higher concordant or better EZ localization than visual analysis, MRI, FDG-PET, and ictal SPECT alone in adults and children with drug-resistant epilepsy (57–59). SISCOM of subcortical structures, that is, ipsilateral basal ganglia, thalamic, and contralateral cerebellar hyperperfusion could provide additional clues for EZ lateralization (60). The combination of SISCOM with other image modalities provides better EZ localization than using it alone. SISCOM plus EEG source imaging (ESI) showed good localization in TLE (61). From a meta-analysis, SISCOM had moderate sensitivity for EZ localization and gave additional information when MRI is negative (62). SISCOM also provides predictive values for post-surgical outcomes. The post-surgical outcome is associated with the extent of the resection site overlapping with SISCOM results (63), and the seizure-free odds ratio was 2.44–3.28 times higher in concordant than in non-concordant SISCOM to the resected region (62). TLE patients with increased perfusion in the contralateral occipital area by SISCOM tend to have less seizure freedom than those without (64). Lobar concordance of SISCOM localization and ictal onset identified on scalp EEG was significantly correlated with the post-surgical outcome in children with TLE (65).

Many other techniques have been recently implemented other than using SISCOM to aid the EZ localization. SPECT reconstruction method that jointly reconstructed ictal and interictal SPECT projection data showed a reduction of noise in the image used for seizure localization than the conventional subtraction method and showed good EZ localization (66, 67). Clearance patterns of ^99m^Tc-ECD are also helpful in evaluating the seizure onset zone in that the seizure onset zone showed slower washout in ictal SPECT but faster washout in interictal SPECT than in contralateral brain regions (68). Scaled subprofile model principal component analysis of ictal brain perfusion SPECT provided independent information from demographic and clinical data for the prediction of post-surgical outcomes in patients with TLE (69).

Quantitative analysis of brain perfusion SPECT with an age-matched normal database was performed much lesser than that of FDG-PET; however, there are several studies with good clinical values that have been published in the last decade. Statistical ictal SPECT coregistered to MRI analysis (STATISCOM) decides statistically significant perfusion changes in epilepsy patients compared with healthy controls. Some studies stated that STATISCOM was superior to SISCOM for EZ localization in MRI-negative epilepsy patients because SISCOM did not compensate for physiologic variance in CBF (70). Interestingly, STATISCOM showed better agreement with video EEG data with no effect of delayed radiotracer injection time, which is in contrast to SISCOM (71). Statistical Parametric Mapping (SPM) software (Wellcome Trust Center for Human Neuroimaging, UCL, London, UK; http://www.fil.ion.ucl.ac.uk/spm), a free and open-source software based on the MATLAB platform (MathWorks, Inc., Natick, MA, USA), is commonly used worldwide for brain functional imaging analysis, including SISCOM and STATISCOM. However, the process of SPM analysis is quite complex and requires some experience. There are several optionally free and commercial software or programs with more simplified processes available. FocusDET, a toolbox for SISCOM analysis, showed low registration error and provided user-editable SPECT-SPECT and SPECT-MRI registration (72). MNI SISCOM is an open-source software graphical desktop application, which provides command line and Python interfaces for users who would like to integrate these features into their own scripts and pipelines (73). Among MIMneuro (MIM Software Inc., Cleveland, OH, USA) analysis, STATISCOM, and SISCOM using SPM, STATISCOM showed the best performance for seizure onset zone localization, followed by MIMneuro and SISCOM, respectively (74). Quantitative analysis of ^99m^Tc-ECD using NeuroGam software (GE Medical System, Segami Corp., Columbia, MD, USA) showed a higher sensitivity for EZ localization than EEG, MRI, or visual analysis (75, 76). Qualitative analysis of ^99m^Tc-HMPAO SPECT and quantitative analysis using SPM to compare patient's image data with a normal template by Z-score map and extract Z_max_ of each voxel with the percentage of voxels with a Z-score higher than 2.5 could provide a diagnosis of nonconvulsive status epilepticus (NCSE) (77).

#### Brain FDG-PET

Brain FDG-PET has been used as a biomarker to assess cerebral glucose metabolism, although not reflecting the whole proportion of glucose metabolism (78). Because FDG-PET scans are performed during the interictal phase, interictal FDG PET is often shortly called FDG-PET. Hypometabolism on FDG-PET reflects the functional deficit zone, which usually encompasses EZ. While visual analysis alone is commonly reported to be negative (79, 80), one study showed that the visual re-assessment of FDG-PET could provide the possible functional deficit zone when the previous results were reported as negative (81). Ipsilateral thalamic hypometabolism could provide additional value for lateralization in TLE without hypometabolism in the temporal lobe (82).

Many studies have shown that quantitative FDG-PET provides much more critical information. Quantitative measurement of the standardized uptake value (SUV) of the region of interests (ROIs) is routinely performed. The selection of ROIs as global or a portion of the temporal lobe showed differences in EZ lateralization in TLE with better identification using the global temporal lobe (83). SUV ratio (SUVr) is an intensity normalization of SUV in which the SUV of ROIs is divided by the SUV of reference regions, such as cerebellum gray matter (GM) or the mean intensity of the skull-stripped FDG-PET image. Quantification of hippocampal glucose uptake by SUVr increases the detection of hippocampal sclerosis over conventional visual analysis (84). Quantitative analysis using SPM requires the selection of a threshold with *p*-value and cluster sizes. Using SPM analysis by comparing the patient's image with a controlled database, a combination of the uncorrected *p-*value of < 0.005 and a cluster size of more than 200 yielded the best EZ localization in visually negative FDG-PET studies (79). In TLE, SPM analysis comparing patients' images with age-matched controls showed that there were larger areas of extratemporal hypometabolism in the post-operative seizure-recurrence group compared with the seizure-free group, for example, hypometabolism at the bilateral anterior cingulate and right orbitofrontal in patients with right TLE (85) or ipsilateral insula and contralateral temporal pole (86) or contralateral frontal and thalamic areas (87) in the seizure-recurrence group. On the contrary, the post-operative seizure-free group showed significant hypometabolism restricted to the ipsilateral temporal tip and hippocampal area (87). Distinct epileptic networks in patients with right vs. left mTLE were observed. Patients with right mTLE showed significantly higher rates of contralateral temporal lobe hypometabolism, which might be a predictor of poor post-surgical outcomes (88). SPM analysis of the high sudden unexpected death in the epilepsy risk group showed bilateral medial frontal and inferior frontal hypometabolism (89). SPM with some modifications on data preprocessing or processing, for example, SPM-computational anatomy toolbox and PET-analysis software that allowed observers to modulate thresholds with multiple *p*-values and different cluster sizes in real time, showed significantly higher EZ localization than that obtained with the conventional SPM (90, 91). Block-matching normalization method, where most transformations are computed through small blocks, showed more accurate EZ localization than conventional SPM normalization methods in patients with TLE (92). SPM t-score maps of FDG-PET could be displayed as 3D SPM t-score surface maps using FreeSurfer (http://surfer.nmr.mgh.harvard.edu) and showed good concordance with ECoG for EZ localization (93). MIMneuro software not only characterized brain perfusion SPECT but also cerebral glucose metabolism by using a normal FDG-PET database and could build an in-house normal database in addition to the default western population database (94). The values of the Z-score in different brain regions are displayed in color on the images and in number. In patients with non-lesional TLE, MIMneuro showed moderate agreement with visual analysis with better delineation of small lesions and increased clinicians' confidence in diagnosis (95, 96). Scenium (Siemens software, Knoxville, TN, USA) is another automatic program for comparing patients' images with a group of normal databases and displays the result in the standard deviation on both the image and in number. It proved to be of additional value to visual assessment in extratemporal lobe epilepsy when the visual assessment was negative (80). The age-matched database is important when evaluating pediatric patients because there are age-related variations in regional cortical glucose metabolism asymmetry as left over right in the frontal and right over left in the posterior regions (97). Therefore, an age-matched pseudo-control group to optimize SPM analysis of FDG-PET in pediatric epilepsy was proposed (98). Functional deficit zone in pediatric epilepsy using in-house age-specific FDG-PET templates by linear registration between PET images and pediatric MRI template showed better agreement with the clinical diagnosis than using the in-built adult control dataset (99). A pediatric-age-specific FDG-PET template based on a nonlinear optimization method improved spatial normalization and showed better EZ localization than using the adult template and linear template (100). Three-dimensional stereotactic surface projection (3D-SSP; NEUROSTAT) software is another user-friendly voxel-based brain mapping software for FDG-PET, which was proven to improve EZ detection compared with visual assessment and MRI and its performance is comparable with SPM analysis (101). Hypometabolism of the ipsilateral hippocampus and amygdala on FDG-PET with 3D-SSP could predict a good surgical outcome for patients with mTLE (102). Visual assessment of FDG-PET combined with SPM or 3D-SSP can improve the sensitivity of EZ detection in MRI-negative patients with drug-resistant epilepsy (103).

Several methods for asymmetry measurements are often used for the lateralization or localization of the EZ. AI is the difference between left and right values divided by left and right mean values. AI of SUVr derived from FDG-PET might be used for the evaluation of clinical severity and progress in pediatric epilepsy. Higher AI values were found in those with drug resistance than with seizure remission (104). Benign epilepsy with centro-temporal spikes (BECTSs), which possess a less favorable outcome than typical BECTS, was shown to have higher AI and more hypometabolic regions than typical BECTs on FDG-PET (105). AI-derived from FDG-PET and arterial spin labeling (ASL) or T2 mapping showed a positive correlation for TLE lateralization (106, 107). AI of Z-score derived from FDG-PET/CT, hybrid PET/MR, and ASL showed high agreement in EZ lateralization in patients with MRI-negative drug-resistant epilepsy (108). AI mappings were concordant with clinical data on the lateralization and localization of EZ in patients with drug-resistant epilepsy (109). Delayed PET scan 2.5 h after FDG injection better identified EZ with relatively greater AI than the conventional time scan AI (110). AI of dynamic FDG kinetic parameters that reflected net metabolic flux (Ki) and phosphorylation (k3) in EZ showed larger AI values and better correlation with AI of static FDG-PET SUVr than influx (K1) and efflux (k2) in patients with drug-resistant epilepsy and showed a difference from healthy controls. Hypometabolism of EZ may be related to reduced phosphorylation (111). Hypometabolism asymmetry (HA) is the difference between left and right values divided by the maximum values of left and right. HA calculated from an SUV of the hippocampus to generate the hypometabolism probability profile could be used for lateralizing TLE (112). Percentage metabolism loss (PML) is an HA expressed as a number out of 100. PML from SUV of the hippocampus and BA38 (temporopolar neocortex) could be used for EZ localization in patients with TLE with PML cutoffs of 5.77 and 8.36%, respectively, for the hippocampus and BA 38 to detect TLE with a sensitivity of 72.7% and a specificity of 77.8% (113).

An increasing number of advanced techniques for the lateralization and localization of EZ have been observed recently—some focusing on regional brain abnormality but many more stressed analyzing metabolic brain networks. Voxel-level Z-score maps of the kinetic parameter (K_i_) generated from dynamic FDG-PET with a Z-score cut-off of <-1.65 can be used to detect temporal hypometabolism in TLE patients with normal static PET images (114). In non-lesional extratemporal lobe epilepsy, dynamic FDG-PET-created regional glucose metabolic rate maps showed increased detection rate and confidence in the original visual readings (115). Evaluation of cerebral glucose metabolic networks using graph theoretical or connectivity analysis demonstrated different global and local metabolic connectivity between TLE and healthy controls (116, 117), between left and right TLE (118), and between TLE with and without hippocampal sclerosis (119) and showed association with post-surgical outcome (120, 121). Graph theoretical analysis was also performed to evaluate the alteration of global or local perfusion network between ictal and interictal states using brain perfusion SPECT in TLE (122) and alteration of metabolic network on FDG-PET in insular epilepsy compared with the control group (123). Area restriction of glucose metabolism in FDG-PET combined with diffusion tensor imaging (DTI) showed different patterns of white matter alterations reflecting the variation of network involvement in TLE with hippocampal sclerosis (124) with fewer white matter fibers observed on the ipsilateral side around the glucose hypometabolic region in patients with drug-resistant epilepsy in another study (109). Distribution–divergence-based method, which was used to construct individual metabolic networks from FDG-PET imaging, could predict seizure-free outcomes in patients with TLE with a sensitivity of 75%, a specificity of 92.79%, and an accuracy of 83.59% (125). A statistical framework using Bayesian hierarchal modeling was implemented to classify between surgical outcomes based on profiles of regional FDG-PET hypometabolism as the phenotypic manifestation of a latent individual pathological state and could predict the subgroup of patients with TLE at high risk of post-surgical seizure-recurrence with high cross-validated accuracy (126). Several studies using supervised or unsupervised machine learning classification algorithms, such as decision tree (DT), logistic regression (LR), logistic model tree (LMT), random forest (RF), multivariate pattern analysis (MPA), support vector machine (SVM), multilayer perceptron (MLP), artificial neural network, convolutional neural network (CNN), and XGBoost, were published. Machine learning using FDG-PET with classification algorithms by MLP (127, 128), LR (129), LMT (130), SVM (131), MPA (132), CNN (133), and combined several methods (134) were used for the lateralization of TLE. Machine learning classification algorithms by DT, LR, RF, SVM, and XGBoost using FDG-PET and/or MRI were used for the detection of focal cortical dysplasia (135, 136). Combined clinical EEG and MRI lesion status with the addition of quantitative PET asymmetry features with the RF method outperformed that using qualitative clinical data to predict TLE with the post-surgical outcome (137).

#### Techniques affecting image quality or workflow

Some techniques have been explored for better image quality before analysis of the image can be performed. A study found that different algorithms (maximum a posteriori reconstructions or maximum likelihood expectation maximization reconstruction with resolution recovery) for FDG-PET brain image reconstruction affected GM activity estimation without altering the detection of hypometabolic regions when performing a voxel-based group comparison in patients with epilepsy (138). MRI GM segmentation of FDG-PET dramatically increased the detection of PET hypometabolic areas corresponding to iEEG onset zones than without segmentation. However, this technique had less impact on Ictal-Interictal SPECT Analysis by SPM (139). For hybrid PET/MR, there have been concerns about the accuracy of MR-based attenuation correction (MRAC) for emitted PET signals. However, studies have shown that using optimal MRAC provided equivalent or non-inferior diagnostic accuracy and sensitivity to PET/CT for EZ localization in adult and pediatric patients with drug-resistant epilepsy (140, 141). Different protocols for PET and MRI acquisition for a hybrid PET/MR machine were studied. Acquiring PET and MRI immediately and simultaneously after FDG injection resulted in significantly higher FDG uptake in the whole brain and many brain regions, while there was less effect when acquiring MRI with PET 40 min after FDG injection, and the findings were similar to acquiring PET without MRI at 40 min (142). Finally, combined PET, MRI, EEG-fMRI, and ESI based on data recorded in a single session using an MR-compatible EEG system and a hybrid PET/MR scanner could avoid multiple scanning sessions and improve the workflow in pre-surgical evaluation (143).

#### Combination of image modalities and hybrid PET/MRI

The combination of multiple neuroimaging modalities usually provides better diagnostic performance for the lateralization and localization of EZ than a single image modality, except in one study, which showed that combined quantitative PET with MRI (cortical thickness) and DTI (white matter anisotropy) did not perform better than PET asymmetry (mean, variance, skewness, and kurtosis) analysis alone for TLE lateralization (144). Other studies were positive for SPECT and/or PET with MRI combinations. Combined MRI and SPECT data using Bayesian model averaging and multinomial LR with linearly weighting posterior probabilities of MRI features and SPECT intensity were proven reliable for TLE lateralization, which probably omits the need for invasive EEG monitoring (145). PET interictal subtracted ictal SPECT coregistered with MRI (PISCOM) that used interictal FDG-PET instead of interictal brain perfusion SPECT was higher or not inferior to SISCOM in identifying EZ in both adult and pediatric patients (146, 147). In MRI-negative patients, many techniques were proven helpful in defining EZ, for example, (1) coregistration of PET/MRI or SISCOM/PET/MRI images was proven useful for epilepsy surgery planning (148); (2) The combination of morphometric analysis program for MRI, PET/MRI coregistration, and SPM analysis of FDG-PET showed higher detection rate than each method alone in MRI-negative focal cortical dysplasia (149–151). (3) Combined morphometric analysis program with quantitative PET increased the specificity of EZ localization coupled with the preferable surgical outcome in MRI-negative patients (152). (4) Automatic fusion by Gaussian mixture model of results from FDG-PET, ASL, diffusion-weighted imaging (DWI), STATISCOM, and ESI with high-density scalp EEG (HDEEG) showed highly accurate seizure onset zone localization in patients with MRI-negative drug-resistant epilepsy (153). Combined FDG-PET and magnetoencephalography (MEG) improved the concordance of EZ localization with surgical resection in patients with drug-resistant epilepsy (154). Poorer post-surgical prognosis showed an association of PET hypometabolism extension beyond SEEG sampling in patients with malformations of cortical development (155).

**Figure 3:**
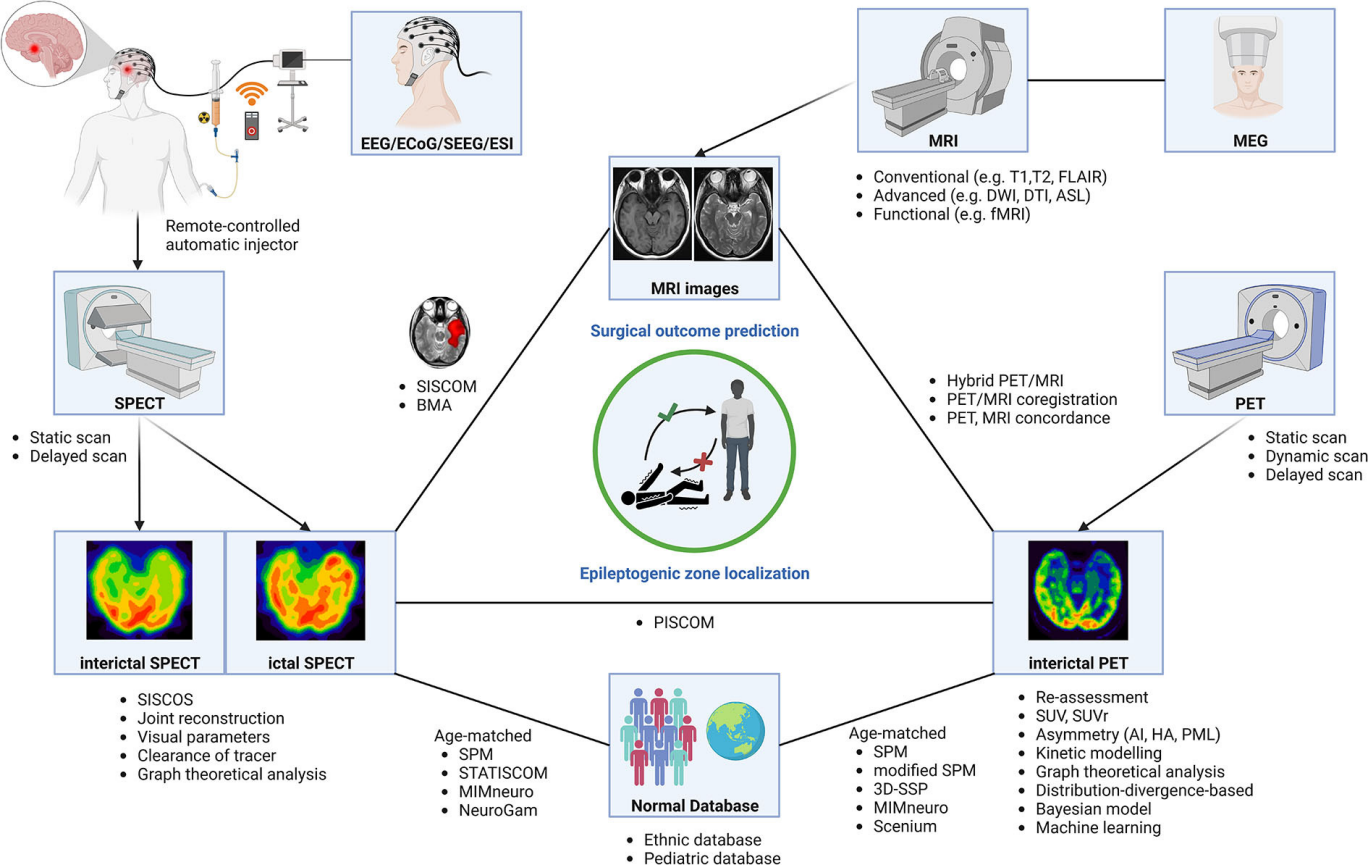
Neuroimaging modalities using various methods and software for epileptogenic zone localization and surgical outcome prediction. AI, asymmetry index; BMA, Bayesian modeling average; DTI, diffusion tensor imaging; DWI, diffusion-weighted imaging; ECoG, electrocorticography; EEG, electroencephalogram; ESI, EEG source image; ETE, extratemporal lobe epilepsy; fMRI, functional MRI; HA, hypometabolism asymmetry; MEG, magnetoencephalography; MRI, magnetic resonance imaging; PET, positron emission tomography; PML, percentage metabolism loss; SEEG, stereotactic EEG; SISCOS, subtraction ictal SPECT coregistered to interictal SPECT; SISCOM, subtraction ictal SPECT coregistered to MRI; SPECT, single-photon emission computed tomography; SPM, statistical parametric mapping; STATISCOM, statistical ictal SPECT coregistered to MRI; SUV, standard uptake value; SUVr, SUV ratio; 3D-SSP, three-dimensional stereotactic surface projection.

A hybrid PET/MR system was recently developed. The system integrates two neuroimaging modalities within one machine allowing synchronous and simultaneous acquisition of anatomical and molecular data. It provided high sensitivity for EZ localization in patients with drug-resistant epilepsy (156–158), which was significantly higher than that of FDG-PET/CT and standalone MRI for EZ localization (159). Combined FDG-PET and ASL from hybrid PET/MR showed high sensitivity, high specificity, and good concordance with histopathology in patients with MRI-negative TLE (160). The technique also significantly increased specificity and PPV in pediatric patients with epilepsy (161). The system could be used for SEEG implantation guidance, and concordance between FDG-PET/MRI and SEEG findings was significantly predictive of a successful surgery outcome (162). Hybrid PET with functional MRI (fMRI) could generate oxygen–glucose index imaging to define the asymmetry for EZ lateralization and localization in patients with TLE (163). The combined fractional amplitude of low-frequency fluctuations (fALFFs) from resting-state fMRI and glucose metabolism from a hybrid PET/MRI system showed higher fALFF/SUVr couplings found in patients who had Engel class IA after surgery than in all other Engel IB-IV (164). Hybrid PET/MR changed surgical decision-making compared with 18F-FDG-PET coregistered with MRI by increasing epileptogenic lesion detection, especially focal cortical dysplasia (165, 166). A concordance analysis method from FDG-PET/MRI could separate operable or implantable from inoperable in drug-resistant epilepsy patients with discordant clinical and diagnostic results or negative-MRI results (167). The overall methods and software for the localization of EZ and prediction of surgical outcomes in the field of PET and SPECT are shown in Figure 3, Table 2.

**Table 2:** Overall methods and software for EZ localization and surgical outcome prediction.

**Methods**	**Data comparison**	**Evaluation**	**Subjects**	**Main objectives**
Visual analysis	Intra-patient interictal and ictal SPECT	Parameters (MP, MC, ME)	ETE	Localization
		Contralateral central structures	TLE, ETE	Prediction
	Individual interictal PET	Re-assessment	TLE, ETE	Localization
Visual (with SUV)	Individual interictal PET	Ipsilateral thalamic area	TLE	Lateralization
Tracer kinetics				
- Kinetic modeling	Individual interictal PET	Kinetic and Z-score maps	TLE, ETE	Localization
- Clearance	Individual interictal or ictal SPECT	Slow, fast washout	TLE, ETE	Localization
SISCOM (by SPM, FocusDET, MNI-SISCOM)	Intra-patient interictal and ictal SPECT	Z-score maps	TLE, ETE	Localization, prediction
		Subcortical structures	TLE, ETE	Lateralization
		Contralateral occipital area	TLE	Prediction
Joint ictal/interictal reconstruction	Intra-patient interictal and ictal SPECT	Reconstructed images	TLE, ETE	Localization
STATISCOM (by SPM)	NDB vs. Interictal and ictal SPECT	Z-score maps	TLE, ETE	Localization
PISCOM (by FocusDET)	Intra-patient interictal PET and ictal SPECT	Z-score maps	TLE, ETE	Localization
Conventional SPM and modified SPM, e.g., SPM-CAT, PET-analysis software, BM normalization, 3D SPM t-score surface maps, optimization	NDB vs. interictal PET	Z-score, t-score maps	TLE, ETE	Localization
		Extra-temporal area	TLE	Prediction
		Contralateral temporal area	Right TLE	Prediction
		Bilateral medial/inferior frontal area	SUDEP	Prediction
	Pediatric NDB vs. interictal PET	Z-score maps	TLE, ETE	Localization
Other quantitative				
- SPECT (by SSM-PCA, NeuroGam)	NDB vs. ictal or interictal SPECT	Z-score maps	TLE, ETE	Differentiation from HC, localization, prediction
- PET (by 3D-SSP, MIMneuro, Scenium)	NDB vs. interictal PET	Z-score maps	TLE, ETE	Localization, prediction
Asymmetry analysis	Intra-patient interictal PET	AI values/maps from SUVr, Z-score, kinetic parameters	TLE, ETE	Differentiation from HC, localization, lateralization
		HPP, HA from SUV of hippocampus	TLE	Lateralization
		PML from SUV of hippocampus and BA38	TLE	Localization
Connectivity analysis				
- Graph theory	NDB vs. ictal or interictal SPECT	Global and local perfusion network	TLE	Localization (EZ, network)
	NDB vs. interictal PET	Global and local metabolic network	TLE, insular epilepsy	Differentiation from HC, differentiation of right and left TLE, TLE with and without HS
- Distribution-divergence-based method	Inter-patient interictal PET	Global and local metabolic network	TLE	Prediction
Bayesian model	Intra-patient interictal, ictal SPECT and MRI	Response-driven model	TLE	Lateralization
	Inter-patient interictal PET	Statistical framework	TLE	Prediction
Machine learning	Intra, inter-patient interictal PET	PET signal with classifiers, e.g., DT, LR, LMT, RF, MPA, SVM, MLP, ANN, CNN, and XGBoost	TLE, ETE	Localization, lateralization, prediction
Combined PET/SPECT/MRI (T1WI, T2WI, DWI, DTI, ASL, MAP, fMRI)/MEG/EEG (ECoG, SEEG, ESI)		Coregistration, concordance	TLE, ETE	Localization (EZ, network), lateralization, prediction

AI, asymmetry index; ANN, artificial neural network; ASL, arterial spin labeling; BA38, temporopolar neocortex; BM, Block-matching; CNN, convolutional neural network; DT, decision tree; DTI, diffusion tensor imaging; DWI, diffusion-weighted imaging; ECoG, electrocorticography; EEG, electroencephalogram; ESI, EEG source image; ETE, extra-temporal lobe epilepsy; EZ, epileptogenic zone; fMRI, functional MRI; HA, hypometabolism asymmetry; HC, healthy control; HPP, hypometabolism probability profile; HS, hippocampal sclerosis; LMT, logistic model tree; LR, logistic regression; MAP, morphometric analysis program; MEG, magnetoencephalography; MLP, multilayer perceptron; MPA, multivariate pattern analysis; MRI, magnetic resonance imaging; NDB, normal database; PET, positron emission tomography; PML, percentage metabolism loss; RF, random forest; SEEG, stereotactic EEG; SISCOM, subtraction ictal SPECT coregistered to MRI; SPECT, single-photon emission computed tomography; SPM, statistical parametric mapping; SPM-CAT, SPM-computational anatomy toolbox; SSM-PCA, scaled subprofile model principal component analysis; STATISCOM, statistical ictal SPECT coregistered to MRI; SUDEP, sudden unexpected death in epilepsy; SUV, standard uptake value, SUVr, SUV ratio; SVM, support vector machine; TLE, temporal lobe epilepsy; T1WI, T1-weighted image; T2WI, T2-weighted image; 3D-SSP, three-dimensional stereotactic surface projection.

## Conclusion

In the recent decade, research in the epilepsy field is still ongoing. The main aims were not only for better delineation of EZ but also for a better understanding of the disease. Collaborations between multidisciplinary teams and a combination of multiple neuroimaging modalities are keys for good EZ localization. Concordant results among each imaging modality usually predict good surgical outcomes. There have been newly emerging radiopharmaceuticals development and increasing indications for their usage. Specific radiopharmaceuticals are still in need of more specific delineation of epileptogenic focus, not only generally, but also for specific epileptic syndromes. Many simple and sophisticated techniques to aid in better EZ identification or revealing the epileptogenic network were implemented. Although many sophisticated techniques have been used as tools to identify epileptic networks, more research is still needed to confirm the usage of these tools in clinical settings. The trend is moving toward the theory of an epileptic network rather than a single epileptogenic focus, which underlies surgically refractory epilepsy. A hybrid PET/MR scanner might avoid multiple scanning sessions and improve the workflow in pre-surgical evaluation. Furthermore, automatic software or more user-friendly programs may ease routine clinical usage. All these developments will potentially result in a rising standard for epilepsy care in the future.

## Data availability statement

The raw data supporting the conclusions of this article will be made available by the authors, without undue reservation.

## Author contributions

CS: wrote the original draft, designed, and created Figures 1–3. ST: reviewed and edited the original draft manuscript and the selection process. CS and ST: conceptualization, performed independently the literature search and data extraction, compared their final report selection, and reviewed the final manuscript. All authors contributed to the article and approved the submitted version.
